# Functional Identification of the *Plasmodium* Centromere and Generation of a *Plasmodium* Artificial Chromosome

**DOI:** 10.1016/j.chom.2010.02.010

**Published:** 2010-03-18

**Authors:** Shiroh Iwanaga, Shahid M. Khan, Izumi Kaneko, Zoe Christodoulou, Chris Newbold, Masao Yuda, Chris J. Janse, Andrew P. Waters

**Affiliations:** 1 Mie University, School of Medicine, Tsu 514-0001, Japan; 2 Leiden Malaria Research Group, Centre of Infectious Diseases, Leiden University Medical Centre, Leiden 2333 ZA, The Netherlands; 3 Weatherall Institute of Molecular Medicine, John Radcliffe Hospital, University of Oxford, Oxford OX3 9DS, UK; 4 Division of Infection and Immunity, Faculty of Biomedical Life Sciences and Wellcome Centre for Molecular Parasitology, Glasgow Biomedical Research Centre, University of Glasgow, Glasgow G12 8TA, Scotland

**Keywords:** MICROBIO, CELLBIO

## Abstract

The artificial chromosome represents a useful tool for gene transfer, both as cloning vectors and in chromosome biology research. To generate a *Plasmodium* artificial chromosome (PAC), we had to first functionally identify and characterize the parasite's centromere. A putative centromere (*pbcen5*) was cloned from chromosome 5 of the rodent parasite *P. berghei* based on a *Plasmodium* gene-synteny map. Plasmids containing *pbcen5* were stably maintained in parasites during a blood-stage infection with high segregation efficiency, without drug pressure. *pbcen5*-containing plasmids were also stably maintained during parasite meiosis and mitosis in the mosquito. A linear PAC (L-PAC) was generated by integrating *pbcen5* and telomere into a plasmid. The L-PAC segregated with a high efficiency and was stably maintained throughout the parasite's life cycle, as either one or two copies. These results suggest that L-PAC behaves like a *Plasmodium* chromosome, which can be exploited as an experimental research tool.

## Highlights

► *Plasmodium* centromere, *pbcen5*, was cloned from the rodent parasite *P. berghei* ► *pbcen5*-containing plasmids were stably maintained during infection and in mosquitoes ► *pbcen5* was used to generate a linear *Plasmodium* artificial chromosome, L-PAC ► L-PAC segregated efficiently and was stably maintained during the parasite life cycle

## Introduction

The centromere in eukaryotic cells plays a fundamental role in the fidelity of chromosome segregation during nuclear division, through its physical association with the kinetochore. Centromere-associated functions include sister chromatid association and separation, microtubule attachment, chromosomal movement, and establishment of heterochromatin and mitotic checkpoint control (Cleveland et al., 2003; Morris and Moazed, 2007; Pluta et al., 1995). The centromere was first identified in the budding yeast *Saccharomyces cerevisiae*: a plasmid containing a yeast centromere was demonstrated to segregate stably during both mitosis and meiosis, providing experimental evidence for the function of the centromere (Clarke and Carbon, 1980). Following this functional identification, a linear DNA construct, including the centromere, telomeric DNA from *Tetrahymena* rDNA termini, and the autonomously replicating sequence, was generated and termed yeast artificial chromosome (YAC) (Murray and Szostak, 1983). YACs can be stably maintained in a linear form in cells during nuclear division and are widely utilized as a vector for transferring large DNA fragments in yeast (Burke et al., 1987). Several artificial chromosomes for other eukaryotes have since been constructed using this approach of combining the same three elements (Harrington et al., 1997; Ikeno et al., 1998). These artificial chromosomes are being used not only as gene-transfer and cloning vectors but also as genetic tools in chromosome function/biology research (Grimes et al., 2004; Okada et al., 2007).

The goal of this study was to generate a *Plasmodium* artificial chromosome (PAC) as a tool to assist in genome and genetic studies. To achieve this goal, we had first to functionally identify and characterize the centromeres of *Plasmodium*. Putative centromeres (PCENs) of the unicellular protozoan parasite *P. falciparum* were previously identified as 2–3 kb A/T-rich regions with no protein-coding potential, based on a sequence comparison between two *P. falciparum* chromosomes (Bowman et al., 1999). After completion of the genome sequence of *P. falciparum*, PCEN regions with similarly high A/T-rich content (>96%) were described for 13 of the 14 chromosomes (Gardner et al., 2002). Further comparisons of *P. falciparum* chromosomes with a whole-genome synteny map of three rodent malaria parasites (RMPs) revealed a high level of synteny between the genomes of different *Plasmodium* species that extends to the location of the PCEN (Kooij et al., 2005). A more recent study located PCENs on *P. falciparum* chromosomes by examining the distribution of etoposide-mediated topoisomerase-II cleavage sites (Kelly et al., 2006). The PCEN locations identified by this study agreed entirely with the locations predicted by whole-genome sequencing and confirmed the presence of A/T-rich domains with a strict size range of 2.3–3.5 kb. Although these studies have provided an insight into sequences and locations of PCENs, experimental evidence is lacking as to whether these highly A/T-rich DNA regions alone can function as *Plasmodium* centromeres. Based on the syntenic location of PCEN, we identified and cloned the PCEN of *P. berghei* chromosome 5 (*pbcen5*) in plasmids. We show that plasmids containing *pbcen5* were both efficiently and stably maintained in transfected parasites during blood-stage multiplication, without drug pressure, demonstrating that *pbcen5* can confer the function of a *Plasmodium* centromere.

In order to generate a PAC, we combined both *pbcen5* and *Plasmodium* telomere sequences into a single plasmid. The third conventional element of a YAC, autonomous replication sequences, has not been identified in *Plasmodium*. However, much *Plasmodium* research has involved plasmid transfections, and despite the varying degrees of segregation efficiency of these plasmids into daughter parasites, they have all been able to replicate inside the parasite, indicating that a variety of DNA sequences can act as *Plasmodium* origins of replication. Telomere sequences of *P. berghei* have been previously identified and cloned (Pace et al., 2000; Ponzi et al., 1985). Evidence has been found that telomere lengthening occurs in *Plasmodium* chromosomes mediated by the activity of *Plasmodium* telomerase enzyme (Figueiredo et al., 2005; Pace et al., 2000; Ponzi et al., 1992). Here we provide evidence that a linear construct, L-PAC, containing both the identified centromere, *pbcen5*, and telomere sequences behaves like a *Plasmodium* chromosome. It segregates with high efficiency during mitosis and meiosis, all in the absence of drug selection pressure, and is stably maintained during the complete life cycle as a few (unconcatemerized) copies. Moreover, we find evidence for telomere elongation, in keeping with the L-PAC being maintained and recognized as a *Plasmodium* chromosome. We discuss the possibilities of using PACs in *Plasmodium* research and how it promises to significantly expand the currently limited set of genetic modification techniques available to understand and manipulate the parasite.

## Results

### Cloning and Sequence Properties of Putative *Plasmodium* Centromeres

**Figure 1:**
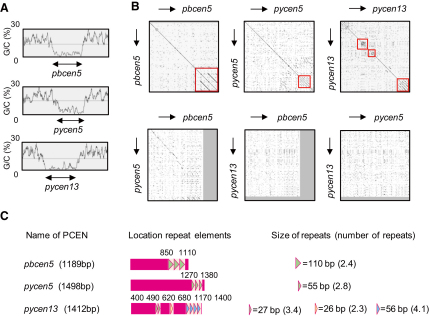
PCENs Are Highly A/T Rich and Consist of Core and Repetitive Regions (A) The DNA AT content of centromeric regions of *P. berghei* chromosome 5 and *P. yoelii* chromosomes 5 and 13 was analyzed using Artemis 10. Arrows indicate the ultra-high A/T-rich regions termed PCENs (*pbcen5*, 1189 bp; *pycen5*, 1498 bp; and *pycen13*, 1412 bp). (B) Dot matrix analysis of the three rodent malaria PCENs using the Dotlet program. In this analysis, only the PCEN regions identified in (A) were used. Panels depict the graphical results of the matrix analysis of the rodent PCENs aligned either against themselves or against the other rodent PCENs. The diagonal line within each analysis represents sequence identity, and parallel lines to the diagonal indicate repetitive regions within each PCEN. Boxes highlight repetitive regions used for further analysis. See also Figure S1A. (C) Lengths and numbers of sequence motifs in the repetitive regions (boxes in B) identified in the dot matrix analyses were determined using the Tandem Repeats Finder program (see also Figure S2 and Table S1). Based on these analyses, the locations, lengths, and numbers of repetitive elements are schematically depicted for the three rodent PCENs analyzed. Each triangle represents a repetitive sequence motif/element. See also Figure S1B and Table S1.

In our previous study, PCENs from chromosomes 5 and 13 of *P. yoelii* were cloned by PCR amplification based on the gene-synteny map of RMP (Kooij et al., 2005). DNA fragments of 4.5 and 4.1 kb were obtained from chromosomes 5 and 13, respectively, which included highly A/T-rich regions predicted as PCENs: *pycen5* (DQ054838.1, 1498 bp, A/T = 97.7%) and *pycen13* (DQ054839.1, 1412 bp, A/T = 98.1%) (Figure 1A). In this study, we amplified the PCEN of chromosome 5 from *P. berghei*. For this PCR we used the same primer pair as was used for *pycen5*, because there were no available sequences from the regions proximate to the PCENs of *P. berghei* based on the RMP gene-synteny map (see the Supplemental Experimental Procedures available online). The amplified 3.8 kb DNA fragment included a highly A/T-rich region, and thus it was predicted to be *pbcen5* (i.e., GU809989, 1189 bp and A/T content of 96.1%; Figure 1A).

To investigate the sequence properties of *pbcen5*, a dot matrix analysis was performed using the Dotlet program (Figure 1B and see also the Supplemental Experimental Procedures). In this analysis, a strict threshold was applied to determine sequence identity, as the sequence complexity of PCEN was low due to its high A/T content. Identity was only recorded if the sequences within a 15 bp sliding box were more than 80% identical. The dot matrix analysis showed that *pbcen5* included a repetitive region indicated by parallel lines in Figure 1B. Furthermore, similar analyses of *pycen5* and *pycen13* as well as 13 annotated PCEN regions from *P. falciparum* (sequence information for PFCEN on chromosome 10 is absent) showed that these PCENs contained one to three repetitive regions. These results suggest that PCENs consist of the nonrepetitive region, termed the “core region,” and the repetitive region, and this general sequence organization is conserved between different *Plasmodium* species (Figures 1B and 1C and Figure S1).

Reciprocal dot matrix analysis between various PCENs showed that sequence identity (indicated by the diagonal line) was found between *pbcen5* and *pycen5* (Figure 1B, the left panel in the lower row). A BLAST2 analysis between these two rodent malaria PCENs showed that they shared homology with a sequence identity of 79%. In contrast, no significant sequence identity was found in reciprocal analyses between the other rodent malaria and *P. falciparum* PCENs (data not shown). Interestingly, sequence identity between *pbcen5* and *pycen5* was restricted to the core regions, indicating that the rates of evolution of the core and the repetitive regions are different.

We further analyzed the possible existence of consensus repetitive motifs within the repetitive regions of the PCENs using the Tandem Repeats Finder program. As can be seen in Figure 1C, Figure S1, and Table S1, different lengths and numbers of repetitive motifs can be distinguished in the various PCENs; however, because of widely divergent sequences, no consensus repetitive repeats were detected (data not shown).

### Segregation of Plasmids Containing a PCEN in *P. berghei* Transgenic Blood-Stage Parasites

During blood-stage asexual multiplication, *Plasmodium* chromosomes are segregated between daughter cells during a process termed schizogony, which differs in several aspects from mitosis in most other eukaryotes. After DNA replication, chromosome segregation occurs without observable chromosomal condensation or breakdown of the nuclear envelope (Aikawa, 1966; Rudzinska, 1969), resulting in polyploid syncytia after several (three to four) rounds of genome duplication. Only in the final step of mitosis when the individual daughter parasites (8–16 merozoites) are formed do the individual nuclei bud off from the syncytium. In contrast to this even segregation of chromosomes between the daughter cells, episomally maintained plasmids (introduced by transfection) segregate unevenly during schizogony, such that several of the daughter merozoites do not receive plasmids during each multiplication cycle (O'Donnell et al., 2001; van Dijk et al., 1997). This uneven distribution leads to a growth disadvantage and is believed to explain why plasmids are rapidly lost when transfected parasites are grown in the absence of drug pressure.

**Figure 2:**
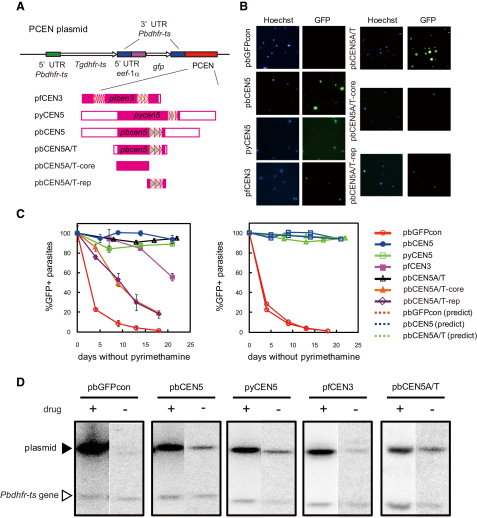
Addition of PCEN to Plasmids Improves Their Segregation Efficiency during *P. berghei* Blood-Stage Multiplication (A) Schematic representation of the various PCEN plasmids. The plasmid contains the *Tgdhfr-ts* selectable marker and the GFP reporter protein under the control of the *eef1αa* promoter. pbGFPcon is a control plasmid without a PCEN. The names of the resulting PCEN plasmids are indicated to the left of the schematic drawings of the cloned PCEN DNA fragments. (B) GFP expression of Hoechst 33258-stained (all parasites) blood-stage *P. berghei* transfected with the various PCEN plasmids, 18–21 days after drug withdrawal. (C) The percentage of GFP-positive parasites during asexual multiplication of blood-stage *P. berghei* that have been transfected with the various PCEN-containing plasmids (left panel). Observed percentage (right panel) of GFP-positive parasites determined by fluorescence microscopy (solid lines) compared to the predicted percentage of GFP-positive parasites (dashed lines), based on the calculated segregation efficiencies of the various plasmids (see also Figure S2). The error bars represent standard error. (D) Southern analysis of the presence of plasmids in the parasites either after 7 days of multiplication in the presence of pyrimethamine (+) or after 18–21 days in the absence of pyrimethamine (−). Blots were hybridized to the *P. berghei* 5′UTR *dhfr-ts* probe, which recognizes the plasmid (black triangle) and the endogenous single *dhfr-ts* genome copy (white triangle, 4.9 kb). The sizes of signals from each plasmid are as follows: pbGFPcon, 9.0 kb; pbCEN5, 12.4 kb; pyCEN5, 13.1 kb; pfCEN3, 11.5 kb; and pbCEN5A/T, 10.4 kb.

To determine whether *pbcen5* contributed to efficient segregation of episomal plasmids during schizogony, we examined the stability of a plasmid including *pbcen5* in blood-stage parasites without drug pressure. As shown in Figures 2B and 2C, the GFP expression in parasites transfected with the control pbGFPcon plasmid lacking PCEN was rapidly lost due to its uneven segregation. However, GFP expression remained stable in the parasites transfected with the plasmid including *pbcen5*, termed pbCEN5 (Figure 2A), and approximately 90% of the parasites with the pbCEN5 plasmid continued to express GFP 21 days after drug pressure had been removed (Figures 2B and 2C). During parasite blood-stage development, we found no growth disadvantage conferred by retaining the pbCEN5 plasmid, as compared to either wild-type or pbGFPcon-transfected parasites. This is based on the observation of comparable courses of parasitemia after infection with standard dose of 10^4^–10^5^ of either wild-type or PCEN-containing parasites. These results clearly demonstrate that the pbCEN5 plasmid can be stably maintained in parasites independent of drug treatment during multiplication, indicating that pbCEN5 segregated evenly into daughter parasites (cells) during asexual blood-stage mitosis.

Similar results were obtained when the stability of a plasmid containing the 4.5 kb DNA fragment including *pycen5* was assessed in the parasite. The percentage of GFP-positive parasites with pyCEN5 was comparable to that of pbCEN5 in the absence of drug pressure as described above (Figures 2B and 2C). Interestingly, the pfCEN3 plasmid was not as stably maintained in blood-stage *P. berghei* as the plasmids containing a centromere of RMP origin. The percentage of GFP-positive parasites decreased to <60% within 21 days (Figures 2B and 2C), although it was higher than that of parasites transfected with the control plasmid pbGFPcon.

To confirm that the PCEN plasmids pbCEN5, pyCEN5, and pfCEN3 were retained as episomes and not integrated into the parasite genome during nuclear replication, we analyzed the genomic DNA of the parasites collected at the start and end of the 18–21 day multiplication period. HindIII-digested DNA was hybridized with a *Pbdhfr-ts* 5′UTR DNA fragment, as this probe recognizes both the endogenous *dhfr-ts* gene (4.9 kb fragment) and the plasmid. As seen in Figure 2D, only the endogenous *dhfr-ts* gene and the plasmid were detected in all parasite populations, both at the beginning and at the end of the multiplication period, indicating that the plasmids were not integrated into the parasite genome but were maintained in episomal form in the parasite nuclei. These results were supported by plasmid rescue experiments in which we were able to recover intact plasmids from all of the parasite populations (data not shown).

Using the percentages of GFP-expressing parasites at the end of the 18–21 day period, we calculated segregation efficiencies for each of the plasmids, based on the assumption that during schizogony the parasite nuclei undergo four nuclear divisions, resulting in the production of 16 daughter nuclei over a 24 hr period (see the Experimental Procedures). The segregation efficiencies of the plasmids pbCEN5, pyCEN5, pfCEN3, and pbGFPcon were calculated as 99.9%, 99.9%, 99.3%, and 93.9% per nuclear division, respectively. When the predicted percentages of GFP-positive parasites were plotted on the basis of the calculated segregation efficiencies over time, it was seen that these percentages fit closely with the observed percentages of GFP-positive parasites (Figure 2C and Figure S2).

Southern analysis of plasmid presence indicated that in parasites maintained in the presence of pyrimethamine, the hybridization signal of the pbGFPcon plasmid was significantly stronger than that of the PCEN plasmids (Figure 2D), indicating that the former plasmid is maintained at a higher number of copies in *P. berghei* (Figure 2D). Comparison of signal intensities for DNA extracted from parasites transfected with the various plasmids and maintained under drug pressure revealed that the pbGFPcon plasmid copy number is on average 28.3 (SE ± 0.3; Table 1), whereas the PCEN plasmid copy numbers are significantly lower (i.e., pbCEN5, 8.0 ± 0.4; pyCEN5, 6.6 ± 0.3; and pfCEN3, 5.4 ± 1.6 copies per parasite; Table 1). After removal of drug pressure, the PCEN plasmid copy number 21 days later was reduced by 2- to 4-fold for the RMP centromere-containing plasmids and >5-fold for pfCEN3. However, there was a strong reduction (>47-fold) in the pbGFPcon plasmid copy number (Table 1). These results indicate that the PCEN elements not only confer greater segregation efficiency to the plasmids but also maintain their low copy number independent of drug treatment.

### Only the Highly A/T-Rich Centromeric Sequences Are Required to Confer High Segregation Efficiency to Plasmid in Blood-Stage Parasites

We next examined whether only the highly A/T-rich region (consisting of both the core and repetitive regions) predicted to be *pbcen5* was sufficient to produce high segregation efficiency. First, the amplified 1.4 kb fragment including *pbcen5* (1189 bp) was cloned into the plasmid, thereby creating the plasmid pbCEN5A/T (Figure 2A). The pbCEN5A/T plasmid was introduced into *P. berghei*, and its stability during mitosis in the absence of drug pressure was examined. Indeed, pbCEN5A/T was stably maintained during mitosis of asexual blood-stage parasites similar to the pbCEN5 plasmid, indicating that only the A/T-rich region (i.e., *pbcen5*) was required to confer high segregation efficiency to episomal plasmids (99.9%) (Figures 2B and 2C). Southern analysis using DNA isolated from the transfected parasites showed that pbCEN5A/T was maintained as an episomal plasmid throughout parasite multiplication and, further, that a low copy number was maintained both in the presence and absence of the drug (Figure 2D, Table 1). These results demonstrate that plasmids containing only the highly A/T-rich region, consisting of the core and repetitive regions, behave essentially the same as the other RMP PCEN plasmids.

To determine the minimal functional element of *pbcen5*, we generated two additional plasmids that contained either only the core (nonrepeat) or only the repeat sections of the A/T-rich region of *pbcen5*, designated as pbCEN5A/T-core and pbCEN5A/T-rep, respectively (Figure 2A). In parasites transfected with these plasmids, the percentage of GFP-expressing blood-stage parasites gradually decreased in the absence of drug pressure (Figures 2B and 2C): segregation efficiencies were 97.7% for pbCEN5A/T-core and 97.3% for pbCEN5A/T-rep. These efficiencies were higher than that of the control pbGFPcon plasmid but lower than those of the plasmids containing the complete A/T-rich region. These results indicate that the entire A/T-rich region is the minimal functional unit of *pbcen5* necessary to permit efficient segregation and maintenance of plasmids during asexual blood-stage mitosis.

### Plasmids Containing PCEN Elements Are Stably Maintained in Parasites during Meiosis and Mitosis inside the Mosquito

**Figure 3:**
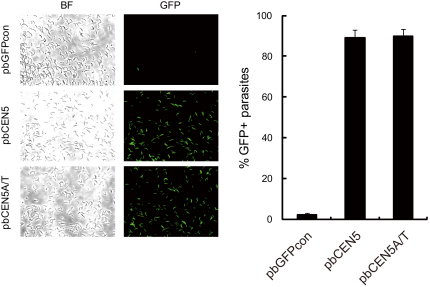
Retention of PCEN-Containing Plasmids in Parasites after Mosquito Passage (Left panel) The GFP expressions in salivary gland sporozoites containing pbCEN5, pbCEN5A/T, or the control pbGFPcon are shown (BF, bright field image of the same sporozoites). (Right panel) The percentages of GFP-positive sporozoites (right) for parasites transfected with the various PCEN-containing plasmids were determined by fluorescence microscopic analysis. The error bars represent standard error.

In addition to asexual multiplication during blood-stage development, malaria parasites have a sexual phase involving meiosis (within the zygote/ookinete) and additional phases of extensive asexual multiplication in the mosquito host (within the oocysts). During meiotic and mitotic division of parasites in the mosquito host, segregation of chromosomes also occurs without chromosome condensation or breakdown of the nuclear membrane, resulting in polyploid nuclei (polyploid syncytia) containing as few as 4 (i.e., the tetraploid ookinete, after meiosis) to as many as 10,000 haploid genomes (i.e., midgut sporozoites). To analyze the segregation behavior of the PCEN plasmids during meiosis and mitosis in the mosquito, parasites carrying pbCEN5, pbCEN5A/T, or pbGFPcon were transmitted to mosquitoes, and the GFP expression of their salivary gland sporozoites was examined. The results indicated that 89.2% ± 3.6% and 89.9% ± 3.3% of sporozoites infected with parasites carrying pbCEN5 and pbCEN5A/T plasmids, respectively, expressed GFP, whereas only 1.9% ± 1.0% of sporozoites with parasites carrying the pbGFPcon plasmid were GFP positive (Figure 3). A growth disadvantage to parasites carrying either of the PCEN plasmids was not observed, suggesting that retention of these plasmids did not affect on the development of parasites in the mosquito. These results showed that addition of *pbcen5* sequences to plasmids greatly improves segregation and maintenance during both meiosis in zygotes/ookinetes and mitosis in oocysts.

### Generation of a *Plasmodium* Artificial Chromosome

**Figure 4:**
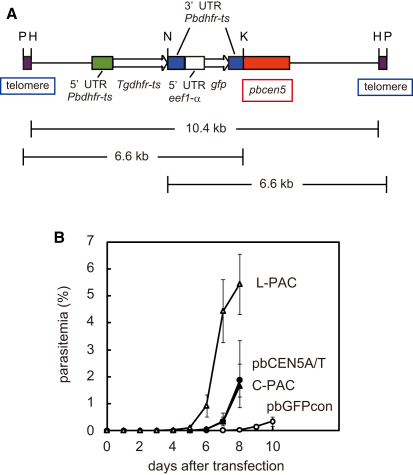
Transfection of Parasites with the Linear *Plasmodium* Artificial Chromosome Construct, Containing PCEN and Telomere Sequences (A) Schematic representation of the L-PAC construct. The construct contains the *Tgdhfr-ts* selectable marker and the GFP reporter protein under the control of the *eef1αa* promoter. Restriction sites for PmeI (P), HindIII (H), NheI (N), and KpnI (K) are shown, which were used for the Southern analysis to determine the size of the DNA fragments digested with each enzyme. (B) The course of parasitemia in mice infected with pbGFPcon (open circle), pbCEN5A/T (closed circle)-, L-PAC (open triangle)-, and C-PAC (closed triangle)-transfected parasites. All parasites (1 × 10^8^) were transfected with 5 μg DNA, and mice were treated with pyrimethamine 1 day after i.v. injection of parasites. The error bars represent standard error.

The results reported above clearly indicate that the region of highly A/T-rich DNA predicted to be a *Plasmodium* centromere did indeed confer the function of a centromere to plasmids during parasite nuclear division. We therefore attempted to construct a PAC consisting of this *pbcen5* centromere as well as *P. berghei* telomeric DNA sequences. Briefly, a DNA insert containing two telomeric fragments, consisting of the previously characterized *P. berghei* telomeric CCCT(A/G)AA sequences (Pace et al., 1987) oriented head to head and separated by an ∼500 bp spacer region, was cloned into the pbCEN5A/T plasmid, resulting in a plasmid designated circular *Plasmodium* artificial chromosome (C-PAC). The linear DNA construct (L-PAC) was generated through linearization of the C-PAC construct using PmeI, which removes the spacer fragment between the two telomeric fragments (Figure 4A). Unexpectedly, the transfection efficiency of the L-PAC was significantly higher than that of the circular plasmids. In multiple independent experiments, the mice injected with L-PAC-transfected parasites developed an parasitemia of ∼1% 6 days after transfection, whereas a parasitemia of ∼1% level was not observed until day 7 or 8 in mice injected with parasites transfected with the same amount of circular plasmid DNA, i.e., the C-PAC or pbCEN5A/T (Figure 4B).

### The *Plasmodium* Artificial Chromosome Is Stably Maintained throughout the Complete Life Cycle

**Figure 5:**
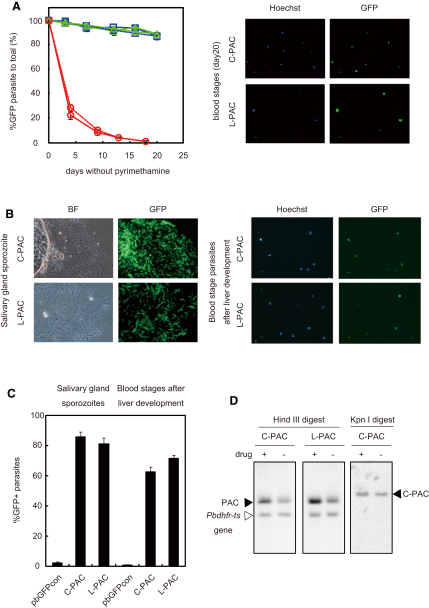
Efficient Segregation and Maintenance of Circular and Linear DNA Constructs Containing Both *pbcen5* and Telomere Sequences (A) (Left panel) The percentages of GFP-positive parasites during blood-stage asexual multiplication of *P. berghei* transfected with the C-PAC (blue), L-PAC (green), or the control plasmid pbGFPcon (red) are shown. The observed percentages of GFP-positive parasites are determined by fluorescence microscopy (solid lines) compared to the predicted percentages of GFP-positive parasites (dashed lines), based on the calculated segregation efficiencies of the various constructs. The error bars represent standard error. (Right panel) The GFP expressions (C-PAC- and L-PAC-containing parasites) of Hoechst 33258-stained transfected parasites (all parasites) were observed by fluorescence microscopy after 18–20 days of asexual multiplication in the blood, without drug pressure. (B) (Left panel) The GFP expressions in salivary gland sporozoite of parasites transfected with the C-PAC or the L-PAC are shown (BF, bright field image of the same sporozoites). (Right panel) GFP expression in the blood stages of parasites transfected with the C-PAC and L-PAC after first completing a passage through the mosquito and multiplication in the liver are shown. (C) Percentage of GFP-positive, variously transfected (C-PAC, L-PAC, and pbGFPcon) salivary gland sporozoites after parasite development in the mosquito, as well as the percentage of GFP-positive blood stages after parasite passage through multiplications in the mosquito and in the liver. The error bars represent standard error. (D) Southern analysis showing the presence of the DNA constructs in blood-stage parasites after either 7 days of multiplication in the presence of pyrimethamine (+) or 20 days in the absence of pyrimethamine (−). Southern analysis was performed on HindIII-digested C-PAC- or L-PAC-transfected parasite DNA with *P. berghei* 5′UTR *dhfr-ts* as a probe, which recognizes both the construct (black triangle) and the endogenous *dhfr-ts* genome copy (white triangle). Based on this result, the copy numbers are estimated and given in Table 1. Southern analysis of C-PAC-transfected parasite DNA digested with KpnI using the *gfp* gene as a probe identified the presence of a single fragment, indicating that the C-PAC is not integrated into the parasite genome.

**Figure 6:**
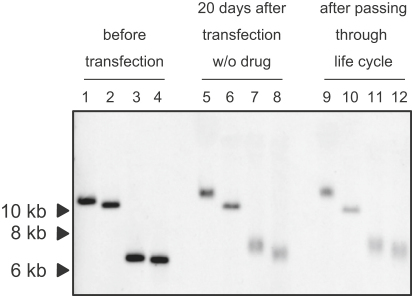
The L-PAC Was Maintained as a Linear Form in the Parasite throughout the Complete Life Cycle Southern analysis to examine the L-PAC construct in transfected parasites after 20 days of multiplication in the blood in the absence of pyrimethamine, as well as in the re-emerging parasite in the blood after mosquito passage and multiplication in the liver. Genomic DNA was digested with HindIII (lanes 6 and 10), KpnI (lanes 7 and 11), or NheI (lanes 8 and 12) and hybridized with the *gfp* gene. In addition, undigested genomic DNA (lanes 5 and 9) was also resolved. Undigested L-PAC (lane 1) and HindIII (lane 2)-, KpnI (lane 3)-, and NheI (lane 4)-digested L-PAC were analyzed under the same conditions.

Segregation and maintenance of the C-PAC and the L-PAC constructs were investigated using the same methodologies as described for the PCEN plasmids. Approximately 85% of the C-PAC- and L-PAC-transfected blood-stage parasites expressed GFP 20 days after withdrawal of drug pressure (Figures 5A and 5B), and their segregation efficiencies at the blood stage were calculated as 99.8% and 99.9%, respectively. To confirm that neither the C-PAC nor the L-PAC was integrated into the parasite genome during replication, Southern analysis was carried out using KpnI-digested genomic DNA for the C-PAC and undigested genomic DNA for the L-PAC. A single 11.5 kb fragment was detected in the KpnI-digested genomic DNA for the C-PAC (Figure 5D), and a single fragment was detected in the undigested genomic DNA for the L-PAC (Figure 6, lanes 5 and 9), demonstrating that neither PAC was integrated into the genome. A similar analysis using HindIII-digested genome DNA for the C-PAC and the L-PAC determined copy numbers of 1.8 ± 0.6 and 2.1 ± 0.6 in drug-treated parasites, respectively, and 1.3 copies for both PAC vectors in the absence of drug treatment (Figure 5D and Table 1). These efficiencies of segregation and copy numbers in the blood stage were comparable to those of the pbCEN5A/T plasmid without the telomeric sequences. Next, we analyzed maintenance of the C-PAC and the L-PAC during mosquito-stage development and found that 83.2% ± 1.6% and 85.5% ± 1.6% of the sporozoites expressed GFP, respectively (Figures 5B and 5C). All of these results demonstrate that the addition of telomeric sequences and linearization did not alter the segregation efficiency and maintenance observed for plasmid pbCEN5A/T during mitosis and meiosis, suggesting that the centromere is able to function within either a circular or a linear DNA construct.

We also examined segregation of the C-PAC and the L-PAC in the dividing forms in the liver. After sporozoites are injected by a mosquito, they migrate to the liver, where they invade hepatocytes. It is in the hepatocytes where they undergo a rapid expansion: one infected hepatocyte can produce several thousand merozoites. For this analysis, it was not possible to estimate the segregation efficiency directly in liver cells; therefore, we assessed the percentage of GFP-positive (i.e., containing either L- or C-PAC) blood-stage parasites as soon as they were patent in the blood after liver stage development. As mentioned above, ∼85% of the PACs-containing sporozoites were GFP positive, and the percentage of GFP-positive infected red blood cells after liver stage development was 62.2% ± 3.4% and 71.2% ± 3.4% for C-PAC and L-PAC, respectively (Figures 5B and 5C). The patent period in mice after infections established with sporozoites containing either C- or L-PAC was comparable to the patent period after infections initiated with the same number of wild-type sporozoites (data not shown). Those results would indicate that despite the rapid expansion in parasite numbers in the liver, plasmids segregated with a high efficiency during the multiple rounds of mitotic division during liver-stage development.

To confirm whether the L-PAC was as an independent linear DNA construct throughout the complete parasite life cycle, we analyzed genomic DNA obtained from blood-stage parasites before and after mosquito transmission. The results of Southern analysis of undigested DNA hybridized with a probe against *gfp* demonstrated that the L-PAC is maintained as an extrachromosomal DNA construct during all stages of the parasite's life cycle (Figure 6). Southern hybridization with the same probe on the same DNA but now digested with the “single cutting” restriction enzymes, KpnI or NheI, revealed only a single band (∼7 kb), which is again consistent with a linear, nonintegrated construct. Interestingly, both in the blood stages after 20 days of multiplication and after mosquito transmission, the size of both the KpnI and the NheI fragments was slightly larger than the expected 6.6 kb fragment of the original L-PAC. Moreover, the total size of the undigested construct was also slightly larger (∼12 kb) than the size of the original L-PAC (10.8 kb). However, the internal fragment of the L-PAC remaining after HindIII digestion, which does not contain the telomeric sequences, was the same size as the original L-PAC. These results would therefore indicate that the increase in size of the undigested construct and the increase in the KpnI- or NheI-restricted fragments results from an increase in size of the two telomeric fragments, indicating that telomere lengthening occurs by addition of telomeric repeats and, further, that the L-PAC telomeres were maintained at a length of up to 1.4 kb.

## Discussion

After completing the sequencing of the *P. falciparum* genome, PCENs were predicted to be within 2–3 kb highly A/T-rich (>96%) gene-free regions. However, despite the recent increase in knowledge about the sequence and location of PCEN, no functional evidence demonstrating that PCEN regions function as centromeres has been reported. In this study, we demonstrated that the A/T-rich region predicted to be the centromere of chromosome 5 of *P. berghei* (*pbcen5*) conferred improved segregation efficiency to episomal plasmids. Transfected plasmids containing *pbcen5* were stably maintained in parasites in the absence of drug pressure. In *Plasmodium*, standard plasmids (e.g., without PCEN elements) are rapidly lost from transfected parasites during asexual multiplication when no drug selection is applied. The stable maintenance of the *pbcen5*-containing plasmids provides strong evidence that the *pbcen5* does indeed function as centromere and that these data constitute the functional characterization of a *Plasmodium* centromere.

The PCEN regions consist of a nonrepetitive region (core) and regions containing repetitive elements, as identified by dot matrix analysis. In an attempt to define a “minimal functional unit” within the *pbcen5* centromere, we analyzed the maintenance of plasmids that contained either only the regions containing repetitive elements or the core sequence. While some increase in segregation efficiency of plasmids is derived from having either one of these elements, high segregation efficiencies were only achieved when both the PCEN repeat and core regions are present, indicating that the entire A/T-rich sequence (i.e., both the core and the repetitive regions) is required for functioning of a centromere. Dot matrix analyses of the other available PCENs from *P. falciparum* and *P. yoelii* indicated a similar organization of all PCENs, consisting of the core and repetitive regions. Despite this conservation of the overall PCEN structure, we did not find conservation in location, sequence, or number of repeats between different chromosomal PCENs, neither within the same genome nor between different species. These results are in large part in agreement with centromere analyses of *P. falciparum* performed by Kelly et al. (Kelly et al., 2006). These observations suggest that the sequence organization and DNA composition of PCEN regions are important to centromere function.

In most strains of *Saccharomyces cerevisiae*, a 6.3 kb plasmid is present, the so-called “2 μm plasmid,” which is replicated by a “rolling-circle” mechanism and is maintained in high copy numbers (50–100 copies/cell) (Broach and Hicks, 1980; Tschumper and Carbon, 1983). The addition of a yeast centromere to the 2 μm plasmid suppresses its rolling-circle replication, resulting in maintenance of only a few copies of the plasmid (one to five copies per cell) (Tschumper and Carbon, 1983). Standard *Plasmodium* plasmids, such as pbGFPcon, also replicate through a rolling-circle mechanism, and these ultimately form large concatameric multimers generated by both intra- and intermolecular recombination. In the present study, plasmids containing *pbcen5* are maintained in low copy numbers (i.e., one to two copies per cell) during blood-stage multiplication, indicating that *pbcen5* also prevents the formation of concatamers by suppressing a rolling-circle replication by the addition of a centromere.

The linear L-PAC construct, containing both the *pbcen5* and telomeric sequences, is stably maintained throughout the complete life cycle of the parasites, indicating that the addition of telomere sequences prevented the degradation of the ends of the linear construct. Attempts to generate transgenic parasites using a linear construct containing *pbcen5* but without telomeric sequences were unsuccessful (data not shown), supporting the importance of telomeric sequences in the maintenance of a linear construct. Our Southern analysis of L-PAC DNA obtained from parasites after successive rounds of replication in both host and vector indicates a lengthening of “telomeric ends” of the L-PAC plasmid and their maintenance within a certain length (1.4 kb). In *P. berghei*, telomere lengthening occurred after the introduction of a short (∼500 bp) telomeric sequence at the end of a chromosome by a targeted terminal deletion event, resulting in a telomere extension to about a length of 1.2 kb (Pace et al., 2000), which is the average length of *P. berghei* telomeres (Dore et al., 1994). This excellent concordance between our present result and previous study suggested that telomere elongation and regulation of its length in L-PAC occurred by the same mechanism observed in parasite original chromosome.

In *P. falciparum*, multigene families encoding clinically important proteins such as the variant antigens PfEMP-1, RIFIN, and STEVOR are located in subtelomeric regions of chromosomes (Gardner et al., 2002). Genes of these multigene families are often expressed in a mutually (or partially mutually) exclusive manner (Scherf et al., 1998). Recent ChIP-on-chip analyses have demonstrated that the Lys9 of histone H3 is trimethylated in both subtelomeric and telomeric regions, and the chromatin structure of these regions is believed to be an important factor in the mutually exclusive gene expression (Lopez-Rubio et al., 2009). In other *Plasmodium* species, the subtelomeric regions contain other multigene families (Janssen et al., 2004), and their transcription may be regulated by similar epigenetic mechanisms that are important in *P. falciparum*. The trimethylation of Lys9 of histone H3 is involved in the regulation of telomeric length. Given that the telomeric length of L-PAC is controlled, it is entirely plausible that L-PAC has a similar chromatin modification that is present in *Plasmodium* chromosomal telomeres. Therefore, this suggested that L-PAC reporter constructs should permit us to examine the mechanism regulating epigenetically controlled gene expression of subtelomerically located multigene families.

Various mutant parasites exhibiting clinically important phenotypes (e.g., drug resistance) have been isolated from field or generated in laboratories thus far. Currently, linkage analysis using progeny clones from genetic crosses between mutant and wild-type parasites is the only way to identify the specific genes responsible for these observed phenotypes. These methods are often complicated, technically challenging, and time consuming. We propose here an alternative approach with gene libraries generated by using L-PAC: genomic DNA or cDNA libraries of mutant parasites are constructed by using L-PAC and then directly introduced into wild-type parasites. The transformed parasites acquiring the mutant phenotype can then be selected by screening (e.g., drug selection), and then target genes incorporated in the L-PAC are identified from the selected parasites. This approach to surveying target genes using the gene library will provide fresh avenues for the analysis of mutant parasites.

Genetic modification of *Plasmodium* using episomally maintained plasmids was first described about 15 years ago (van Dijk et al., 1997), and the use of this method of genetic analysis has contributed significantly to studies of the parasite's biology. However, genetic modification using episomally maintained plasmids has two limitations that make it unsuitable for a number of parasite transformation studies. First, maintenance of the plasmids in parasites requires drug selection to prevent loss of the plasmids during mitosis. Consequently, the use of this technique is mainly restricted to the blood stage and is less suited for studies on parasites in either the mosquito or the liver. Second, episomal plasmids often form undesirable concatamers, resulting in high and variable copy numbers of the plasmid. As a result, the control or estimation of transcription activity of the genes introduced into the plasmid is difficult, and therefore plasmids are not suitable for precisely assessing gene expression levels in reporter assays. These technical limitations can be overcome by using the PAC and PCEN plasmids, since they are stably maintained throughout the life cycle and do not form multimeric concatamers. For example, accurate reporter assays at the mosquito and liver stages will only be achieved by using modified PAC and the PCEN plasmids or equivalents. Indeed, we have recently reported the use of such reporter assays (Yuda et al., 2009). In this study, we generated reporter PCEN plasmids containing several promoter regions (i.e., either containing or not containing *cis*-acting elements) and were able to precisely determine the transcriptional activities of those promoter regions controlled by the ookinete-specific transcription factor.

In conclusion, we describe in this paper the functional characterization of a *Plasmodium* centromere and the generation of PAC that appears to behave like a true *Plasmodium* chromosome. The study not only will expand the number of molecular tools available to malarial research but also sets out a template that should permit the generation of *P. falciparum* PAC.

## Experimental Procedures

### Construction of Plasmids Containing PCEN

The various (complete and partial) PCEN-containing constructs were cloned into the plasmid vector pbGFPcon, which contains a selectable marker cassette encoding the *Toxoplasma gondii* pyrimethamine-resistant *dhfr-ts* gene (*Tgdhfr-ts*) as well as a *gfp* expression cassette under the control of the *P. berghei eef1αa* promoter. pbGFPcon was digested with EcoRI, thereby removing its *d-ssu-rrna* sequence. Subsequently, the PCEN-containing DNA fragments were digested with EcoRI and cloned into the EcoRI-digested pbGFPcon. All PCEN-containing plasmids are schematically shown in Figure 2A.

### Construction of the *Plasmodium* Artificial Chromosome

The DNA fragment containing the two telomeric sequences, oriented head to head with an ∼500 bp spacer region, was digested with HindIII and then cloned into the HindIII-digested pbCEN5A/T plasmid. This resulted in a circular plasmid, termed C-PAC. When we digested the C-PAC with PmeI, removing the spacer region between the two telomeres, the remaining linearized fragment was designated L-PAC.

### Assays to Determine the Efficiency of Segregation and Maintenance of PCEN Plasmids and PACs in Transfected Parasites

Transfected parasites were first maintained for a period of 1–2 weeks in Swiss mice under pyrimethamine drug pressure. After this period, when the parasitemia had reached 5%–10%, the parasites were transferred to naive mice. The parasites were then maintained in these mice and subsequent mice for 18–21 days without drug treatment. During this period, mechanical passage of the parasites was repeated three to five times once the parasitemia in the infected mice had reached 5%–10%. Mechanical passage was performed by intraperitoneal injection of 2–4 × 10^4^ infected erythrocytes. At each mechanical passage, 10 μl of blood was collected from each mouse in 1 ml of culture medium (RPMI1640 medium [pH 7.3], containing 10% fetal calf serum) to determine the percentage of parasites retaining PCEN constructs. This was done by assessing the percentage of infected erythrocytes that were GFP positive using a fluorescence microscope. Specifically, to determine the percentage of GFP-positive parasites, the samples of infected blood were incubated at 37°C for 5 min in the presence of Hoechst 33258 (10 μM final concentration) to stain all parasite nuclei. According to the percentages of GFP-positive parasites at the end of the 18–21 day multiplication period, we calculated the segregation efficiencies of the PCEN-containing constructs in the blood stage based on the assumption that during blood-stage schizogony the parasite nuclei undergo four nuclear divisions, resulting in the production of 16 daughter nuclei over a 24 hr period.

To evaluate segregation and maintenance of the PCEN-containing constructs during mosquito development, transfected parasites were first maintained for a period of 1 week in Swiss mice under pyrimethamine drug pressure. To obtain mosquito-stage parasites, *Anopheles stephensi* mosquitoes (3–4 days old) were fed for 10 min on anesthetized, transgenic parasite-infected mice. Mosquitoes were maintained at 20°C and 80% humidity and fed daily on 10% sucrose. To isolate salivary gland sporozoites, salivary glands were dissected at day 20–22 p.i., collected in ice-cold saline, and homogenized in 200 μl of medium 199. The percentage of GFP-positive sporozoites was determined by fluorescence microscopy.

To analyze segregation and maintenance of the plasmids within the parasites during liver-stage development, 3 × 10^4^ salivary gland sporozoites were injected intravenously into young Wistar rats (3 weeks old). Four to eight days after infection of the rats, the development of blood-stage parasitemia was determined using Giemsa-stained blood smears. At a parasitemia of 5%–10%, tail blood was collected and stained with Hoechst as described above, and the percentage of GFP-positive to Hoechst-stained parasites was determined by fluorescence microscopy.

### Southern Analysis of DNA Collected from *P. berghei* Blood Stages Transfected with PCEN Plasmids and PACs

Genomic DNA was isolated from blood-stage parasites transfected with the various PCEN plasmids and PACs and maintained for 18–21 days without pyrimethamine. Parasite DNA containing the episomal plasmid DNA was digested with HindIII, which cleaves a single site within all PCEN plasmids and two sites in both the C- and L-PAC constructs. Southern hybridization was performed using a 5′UTR DNA fragment of the *P. berghei dhfr-ts* gene as a probe, which is present in all PCEN plasmids and PACs and on chromosome 7 of the *P. berghei* genome. The hybridization intensity of the signal in each sample was quantified using Quantity One software (Bio-Rad). By comparing the intensity of the hybridization signal for the plasmid and the hybridization of the probe to the endogenous *dhfr-ts* gene in the genome, the copy number of each plasmid was determined. In addition, to examine the integration of the C-PAC, genomic DNA was digested with KpnI, which cleaves at a single site within the C-PAC. The digested DNA was processed and analyzed by Southern analysis as described above.

Genomic DNA from parasites transfected with the L-PAC was collected both from blood stages, as described above, and from blood stages after mosquito transmission. This genomic DNA from parasites transfected with the L-PAC was digested with either HindIII, which recognizes two sites within the L-PAC, or with NheI or KpnI, which each recognize a single site within the L-PAC. Hybridization was performed using a fragment of the *gfp* gene as a probe (present exclusively in the L-PAC plasmid), which specifically recognized fragments from the L-PAC vector.

## Figures and Tables

**Table 1:** The Copy Number of PCEN Plasmids and PAC in Parasites

	+Drug	−Drug
pbGFPcon	28.3 (0.3)	0.6 (0.1)
pbCEN5	8.0 (0.4)	1.9 (0.1)
pyCEN5	6.6 (0.3)	2.8 (0.3)
pfCEN3	5.4 (1.6)	0.9 (0.1)
pbCEN5A/T	2.1 (0.3)	1.3 (0.4)
C-PAC	1.8 (0.6)	1.3 (0.1)
L-PAC	2.1 (0.6)	1.3 (0.1)

Copy numbers of PCEN plasmids, C-PAC and L-PAC were calculated from the ratio of intensities obtained from Southern analysis as shown Figures 2D and 5D. The standard errors are indicated in the parenthesis.

## Supplemental Information

Document S1. Two Figures, One Table, and Supplemental Experimental Procedures
