# Functional Identification of the *Plasmodium* Centromere and Generation of a *Plasmodium* Artificial Chromosome

**DOI:** 10.1016/j.chom.2010.04.011

**Published:** 2010-05-20

**Authors:** Shiroh Iwanaga, Shahid M. Khan, Izumi Kaneko, Zoe Christodoulou, Chris Newbold, Masao Yuda, Chris J. Janse, Andrew P. Waters

(Cell Host & Microbe *7*, 245–255; March 18, 2010)

In the author attributions of the above article, the affiliation of Andrew P. Waters was listed as Leiden University Medical Centre and present address as the University of Glasgow. In fact, both institutions should have been listed as affiliations, indicating that contributions to the production of the manuscript had been made at both locations. Any inconvenience caused by this error is regretted. This has been corrected in the original article online.

