# Supplementary material for: Functional Identification of the Plasmodium Centromere and Generation of a Plasmodium Artificial Chromosome
Source: Cell Host Microbe. 2010 Mar 18;7(3):245–55. doi: 10.1016/j.chom.2010.02.010 (PMC2996609; doi:10.1016/j.chom.2010.02.010)
Supplement: Document S1. Two Figures, One Table, and Supplemental Experimental Procedures [file mmc1.pdf]

Cell Host & Microbe, Volume *7*

Supplemental Information

Functional Identification of the *Plasmodium* Centromere and Generation of a *Plasmodium* Artificial Chromosome

Shiroh Iwanaga, Shahid M. Khan, Izumi Kaneko, Zoe Christodoulou,
Chris Newbold, Masao Yuda, Chris J. Janse, and Andrew P. Waters

INVENTORY OF SUPPLEMENTAL INFORMATION

SUPPLEMENTAL FIGURES AND TABLE

1. Figure. S1, related to figure 1. Sequence analysis of PCENs of *Plasmodium falciparum.*
2. Figure. S2, related to figure2. Observed and Predicted Retention of the Variously Transfected PCEN Plasmids
3. Table S1, related to figure 1 and S1. Consensus repetitive motif in each PCEN.

SUPPLEMENTAL EXPERIMENTAL PROCEDURES

1. Computational Analysis of PCEN Regions
2. Cloning of Putative Centromere of *Plasmodium spp.*
3. Cloning of Telomeric Sequences of *P. berghei*
4. Transfection of PCEN Plasmids and PACs into Blood Stages of *P. berghei*

Supplemental Figure S1

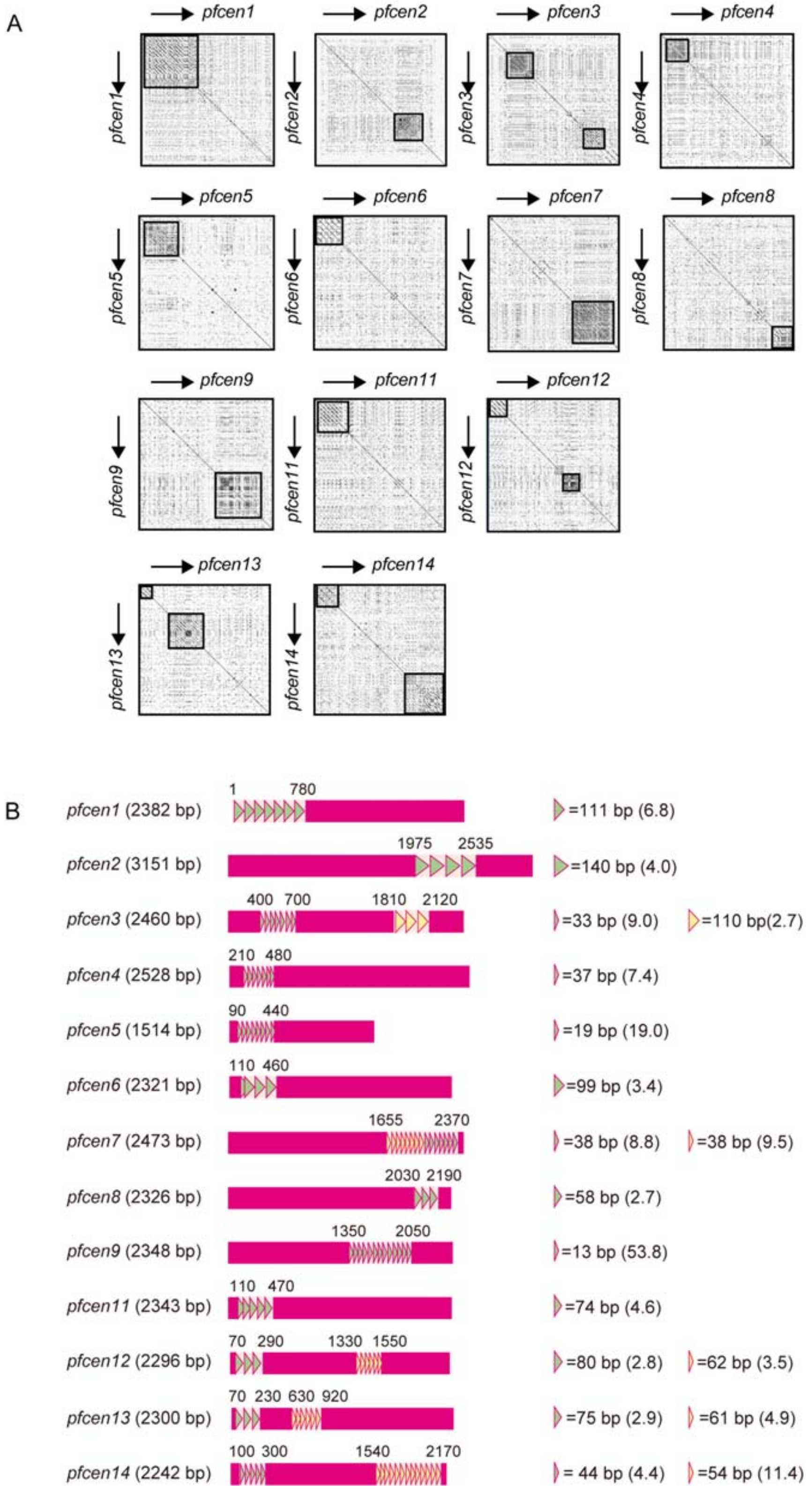

Figure S1, related to Figure 1

Sequence Analysis of PCENs of *Plasmodium falciparum.*

A: Dot matrix analysis of the PCENs of *P. falciparum* using the DOTLET program. In this analysis, only the ultra high AT-rich regions (the putative PCENs) of *P. falciparum* were used. Sequences were obtained from PlasmoDB (http://www.plasmodb.org/). The lines parallel to the central diagonal line indicate repetitive regions in each PCEN. Location, size and number (between brackets) of repeat sequences in *P. falciparum* B: Location, size and number (between brackets) of repeat sequences in *P. falciparum.* PCENs identified by dot matrix analysis using DOTLET as shown in Figure S1A. The repetitive motifs were identified using the TANDEM REPEAT FINDER program.

Supplemental Figure S2

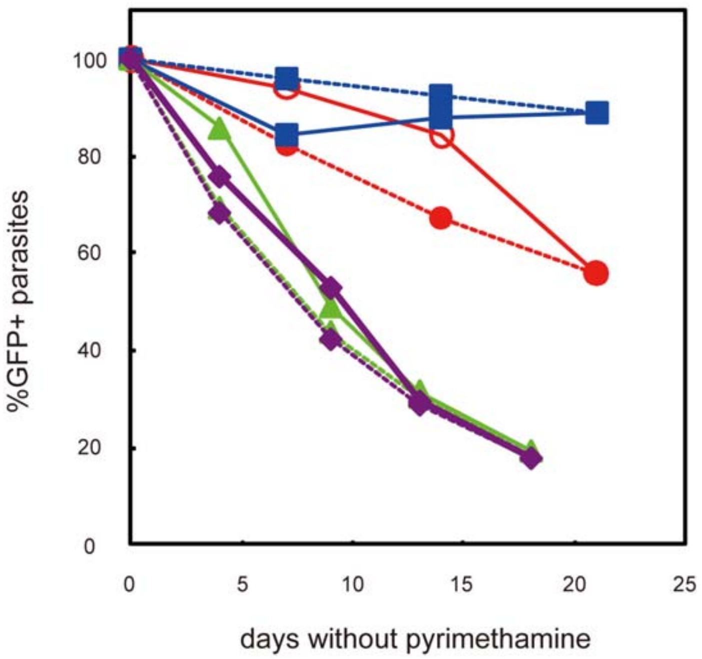

Figure S2, related to Figure 2

Observed and Predicted Retention of the Variously Transfected PCEN Plasmids

The observed and predicted retention of the variously transfected PCEN plasmids, during the course of *P. berghei* blood stage infection (in the absence of drug pressure), based on the percentage of GFP-positive parasites. The observed percentage of GFP-positive parasites was determined by fluorescence microscopy (solid lines) and the predicted percentage of GFP-positive parasites

(dashed lines), based on the calculated segregation efficiency of various plasmids. The error bars represent standard deviation. Calculated segregation efficiencies were: pfCEN3 (red, circle); 99.30%, pYCEN5 (blue, square); 99.86%, pbCEN5A/T-core (green, triangle); 97.65%, pbCEN5A/T-rep (purple, diamond); 97.30%.

Table S1, related to Figure 1 and Figure S1

Consensus repetitive motif in each PCEN.

PCEN	Repetitive sequence motif	Length of repetitive motif (bp)	Copy no.	Alignment score
pfcen1	ATAAATAAAATATTAAATTAAATAAATAAAATATATATAATTAAATAAATAAATAATATATTAAATAAATAAAATATTATATTAAATAAATAAAATAAAATATTAAAAAAT	111	6.8	508
pfcen2	TTTAATTAATTTATTTAATATATTAATTTATTTTTATTTTATTTATTTATTTAATATATTATTTATTTTTATTTTATTTATTTATTATTTAATTTATTTATTTAATATTTTAATTTATTTTATTTTATTTATTTAATTAT	140	4	579
pfcen3-1	ATATATTATTTATTAAAATAAAAATAAATTAA	33	9	569
pfcen3-2	ATTTATTATATTTTAATTAATTTATAATATATTATATTTTAATTAATTTATAATATTTATTAAATTTATTTTAATTAATTAAATAATATAATTAAATTTAATTATATAAC	110	2.7	358
pfcen4	AATTAATTAAAATAAAATAATAATTATATAATATATA	37	7.4	484
pfcen5	TTAATTAAATAAATAATAA	19	19	290
pfcen6	TAATATTAAATTATTTAATTATAATTAAATTAAATATAATAAAAATAATAAATAAAGTAATAAATAATTATATTTAATTAAATAAAATTAAATTAAATA	99	3.4	462
***pfcen7*a**	1:ATATATATATTTAATTATTATTTTAAATTATTTAATTA	38	8.8	430
	2:TATTTAATTAATAAATATATTTAATTATTATTTTTAAT	38	9.5	381
pfcen8	TTAATTAATTAATTATATTTATATTTTAATTAATTAATTAATTATATTAATATTTTAA	58	2.7	222
pfcen9	TTAATTAATTTAT	13	53.8	340
pfcen11	TATTATTTATAATAATAATAAATTATATAAAAATATATATTATTTATATATTTAATTTAATTAAATAAAATATA	74	4.6	582
pfcen12-1	AAAAATAATAATATTTATTATTATATAATATATTTAATTAAATTAAATTTATTATTTAATTAATTAATTTAAAATAAAAT	80	2.8	414
pfcen12-2	ATTATATATATATTTAATTTATTTTATATATATTTAATTTAATTATATATATATTTAATTTA	62	3.5	277
pfcen13-1	TATAAAATAAATTATAATATAAAAAATATATATTAATTATTAAATTAATATTAGAATTTAATTAAATTAAATTAA	75	2.9	352
pfcen13-2	TAAATATAATTAATAATATTATATTAAATATTAAAATAAATAAATAAATTAATTAATTATT	61	4.9	345
pfcen14-1	ATTATTATTTAATATTATATAATATAATTTAATAAATATAAAAT	44	4.4	194
pfcen14-2	TTTATTTATTTAATATTATTTTTATTTATTTATTTTATTATTTTATTAATTAAT	54	11.4	548
pycen5	TATTAATTATTTATATTTATTTTAATATAACCATTTAATTTAATTTAAATATAAT	55	1.9	201
pycen13-1	ATTAAATAATAAAATAATAATATATTA	27	3.4	166
pycen13-2	ATTAAATTAATTAATAAATATTATAT	26	2.3	122
pycen13-3	TTATTATAATTAAAGTAATATAATTAATTAATTATTATTTTTAAAATATATAATTA	56	4.1	289
pbcen5	TTAATTAATATATTAATTATTATATTAGTTTATATTTAAAATATTATATTATTAATTATTATTTAATTATTAATTTTAATATATAAATTATTTATATTAAATATTATTAT	110	2.4	513

Top-scoring sequences were shown in TABLE. a: Two consensus sequence motives were found in the one repetitive region of *pfcen7*.

SUPPLEMENTAL EXPERIMENTAL PROCEDURES

Computational Analysis of PCEN Regions

Sequence analysis of the PCENs was carried out using Artemis 10 software. The following DNA sequences were used: 1) Putatively annotated PCENs of *P. falciparum*, downloaded from PlasmoDB (http://plasmodb.org/plasmo/), 2) existing *P. yoelii* DNA sequences from chromosomes 5 and 13 containing the putative centromeres, including the 4.5 kb fragment containing *pycen5* (GenBank accession no. DQ054838.1) and the 4.1 kb fragment containing *pycen13* (GenBank accession no. DQ054839.1), retrieved from GenBank (www.ncbi.nlm.nih.gov/sites/entrez?db=nucleotide), and 3) the 3.8 kb DNA sequence containing *pbcen5* (GenBank accession no. GU809989). These DNA sequences were analyzed using Artemis 10, their respective AT contents were analyzed using a scanning window (120 bp), and the output was visualized graphically. The ultra-high A/T-rich regions within each query sequence were identified using base composition graphics and termed the putative *Plasmodium*

centromeres (PCENs). To identify repetitive regions within the PCENs, dot matrix analyses of PCENs were carried out using Dotlet with a window size of 15 bp. In these analyses, sequence identity was only recorded if the sequence identity within the 15 bp window was > 80%. The presence and locations of sequence elements in each repetitive region were identified using the program Tandem Repeats Finder. The sequences and arrangements of any repetitive regions within the various PCENs were characterized using the default settings.

Cloning of Putative Centromere of *Plasmodium spp.*

P. berghei ANKA, *P. yoelii* 17NL, and *P. falciparum* 3D7 strains were used for cloning of the *Plasmodium* putative centromere regions. DNA fragments including the putative centromeres of chromosome 5 of *P. berghei* (*pbcen5*) and chromosome 5 of *P. yoelii* (*pycen5*) were amplified using the same primers, P1: 5′-TCATGTGATGTTTACAACTGTGTC-3′ and P2: 5′-AAAAATGTAATTGCCAAAAAGG-3′, with genomic DNA as a template, resulting in fragments of 3.8 kb and 4.5 kb, respectively. The DNA fragment

containing the putative centromere of chromosome 3 of *P. falciparum* (*pfcen3*) was amplified by a two-step PCR procedure. First, a fragment was amplified using the primer pair P3: 5′-TAATATACAAATATAAATCATGC-3′ and P4: 5′-ATAACATATAATAAATTAATTAGC-3′ from genomic DNA. Next, a DNA fragment of 2.5 kb including *pfcen3* was amplified with the primers P5: 5′-ATATAAAGCTTAGTATTATTTATATG-3′ and P6: 5′-TAGGTACCAAATAATAAATACACATC-3′, using the first-step PCR fragment as template DNA. In addition, we amplified a 1.4-kb fragment including *pbcen5* (1189 bp) with the primer set P7: 5′-TAGACAAACAAAATGATTATGATACAAATAC-3′ and P8: 5′-TTATAGGGTTATATTTTATAAGAAATAAGG-3′, using the 3.8-kb fragment as template DNA. Two truncated forms of *pbcen5* termed *pbcen5-core* (973 bp) and *pbcen5-rep* (460 bp), were amplified by PCR using the primer pairs P9: 5′-GCTTATATATCAATTAATAATTATTATTATGC-3′ and P10: 5′-TAGACAAACAAAATGATTATGATACAAATAC-3′ for *pbcen5-core*, and P11: 5′-CGATAATAATAATTATTAATTGATATATAAGC-3′ and P12:

5′-TTATAGGGTTATATTTTATAAGAAATAAGG-3′ for *pbcen5-rep*. All amplified PCR fragments were cloned into the pCR2.1-TOPO vector (Invitrogen).

Cloning of Telomeric Sequences of *P. berghei*

We used an existing DNA fragment containing telomeric sequences of *P. berghei* (ANKA strain) that had previously been cloned into pUC19 . To introduce restriction sites at both ends of the telomeric fragment, PCR was performed with the primer pair P13: 5′-CACCGAAACGCGCGAGTTTAAACCC-3′ and P14: 5′-AAGCTTGCGGCCGCTGACCATGATTACGAAATCCC-3′, resulting in a 0.2-kb telomeric DNA fragment with newly introduced *Pme*I and *Hind*III restriction sites. The amplified fragment was cloned into the pCR2.1-TOPO vector twice and oriented in a 'face-to-face' arrangement with an approximately 500-bp intervening spacer region containing a *Pme*I restriction site, resulting in pTopo-TEL-TEL.

Transfection of PCEN Plasmids and PACs into Blood Stages of *P. berghei*

All PCEN plasmids used for transfection were purified using a Qiagen Plasmid Midi kit (Qiagen). The L-PAC was generated by *Pme*I digestion, as described above. Five micrograms of each DNA construct were transfected into *P. berghei* (ANKA strain) blood-stage schizonts using standard Amaxa transfection technology, as described previously . Transfected parasites were injected into Swiss mice (~20 g) and selected by pyrimethamine treatment of the animals. Each construct was used for at least two separate transfections to generate two independent transfectant parasite lines.
